# A Multi-Modal Fusion Network of Visible–Ultraviolet Features for Detecting Aero-Engine Blade Microcracks

**DOI:** 10.3390/s26165280

**Published:** 2026-08-20

**Authors:** Xin Wang, Caizhi Li, Xiaolong Wei, Weifeng He, Zhigao Wang, Yizhen Yin, Wei Liu, Ligang Wang

**Affiliations:** 1 National Key Lab of Aerospace Power System and Plasma Technology, Xi’an 710038, Chinaweixiaolong9s@126.com (X.W.);; 2 School of Mechanical Engineering, Xi’an Jiaotong University, Xi’an 710049, China; 3 State-Owned Sida Machinery Manufacturing Company, Xianyang 712201, China; 4 AVIC Shenyang Aircraft Company Limited, Shenyang 110850, China

**Keywords:** aero-engine blades, microcrack, fusion, object detection, deep learning

## Abstract

Detecting microcracks in aero-engine blades is paramount for ensuring flight safety, yet conventional inspection methods exhibit significant limitations in precision, efficiency, and applicability. This paper proposes a multi-modal fusion detection network for blade microcracks—VUFNet (visible and ultraviolet multi-modal fusion detection network)—which accurately identifies blade microcracks by integrating visible and ultraviolet image features. First, the blade surface undergoes chromic acid anodisation to enhance microcrack features. Subsequently, visible and ultraviolet light illumination is applied to construct a microcrack dataset. VUFNet employs dual backbone networks to extract visible and ultraviolet features. It incorporates CSPSTR (cross-stage partial bottleneck with swin transformer) to optimise feature extraction capability, and VUFM (visible–ultraviolet multi-modal adaptive fusion module) to deeply fuse visible and ultraviolet features. Finally, MFConv (multi-branch fusion convolution) enhances detection accuracy in microcrack target regions. On the held-out test subset acquired from a single compressor-blade type under a fixed laboratory imaging protocol, VUFNet achieved mAP_0.5_ and mAP_0.5:0.95_ values of 96.8% and 85.5%, respectively, with a detection speed of 52.7 fps. These results demonstrate improved within-domain detection performance over the evaluated baselines. Ablation studies further validate the synergistic optimisation effect of VUFM, CSPSTR, and MFConv on model performance.

## 1. Introduction

As the power system’s core aerodynamic component, the aero-engine blade’s structural integrity directly determines the engine’s thrust-to-weight ratio, fuel efficiency and safety margin. In the manufacturing process, inherent material defects, process deviations, structural design defects and other factors can induce the emergence of surface microcracks, seriously affecting the qualification rate of the blade. During the engine’s service, inhaling foreign materials, such as sand and metal particles, is inevitable. These complex materials impact the blade surface at high speed, forming a complex multi-axial stress field in the impact area, accompanied by micron-level cracks or notch defects. These initial types of damage make it very easy to induce the emergence of microcracks which expand under the joint action of harsh service environment and cyclic loads, seriously affecting engine performance and flight safety [1,2]. Therefore, it is essential to ensure the detection of microcracks on aero-engine blades and determine whether they meet the relevant standards. This initiative is of inestimable value for preventing potential failures, extending engine life, and ensuring flight safety.

Traditional methods for detecting microcracks in aircraft engine blades primarily include visual inspection [3], vibration testing [4], ultrasonic testing [5], penetrant testing [6], and radiographic testing [7]. However, each method has significant limitations: visual inspection is simple and low-cost but struggles to detect microcracks; vibration testing heavily relies on experience and faces challenges in locating defects; ultrasonic testing offers high sensitivity but requires specialised equipment and skilled technicians; penetrant testing can only detect surface defects and has limited effectiveness; and radiographic testing faces high costs and radiation risks. As such, each traditional method has its trade-offs in terms of detection accuracy, applicability, operational convenience, and cost-effectiveness. Therefore, developing a microcrack detection method that combines high efficiency and high precision holds significant engineering application value.

Xu et al. [8] developed a spin hall magnetoresistive sensor to make eddy current detection no longer dependent on demodulation or phase-locking techniques and to enable the classification of a wide range of metal cracks. However, the principle’s complexity and the signal recognition place high demands on the operator. Lee et al. [9] used ultrasonic testing to detect fatigue cracks in the shank holes of the first-stage blades of a J85 engine compressor. They utilised a model-assisted probability of detection (MAPOD) process to assess NDT’s fatigue crack detection capability quantitatively. This study showed that the ultrasonic testing method can effectively detect blade cracks but has problems with the non-intuitive display of cracks and complicated operation techniques. Mevissen et al. [10] developed an ultrasonic-stimulated thermography test system to detect cracks in turbine blades. This test system can quickly and visually display blade cracks, but it requires expensive equipment and is not easy to deploy. Shang et al. [11] used an industrial borescope to perform in situ damage measurements on aero-engine blades. This study showed that the industrial endoscope can effectively detect internal engine blade damage, but it has the problem of having difficulty in detecting microcracks. Xie et al. [12] proposed a blade-damage-monitoring method based on the shaft’s random vibration frequency-domain statistical index. This method can infer the blade damage condition by monitoring the blade vibration signal, but it cannot obtain the damage location. Wang et al. [7] used X-rays to detect aero-engine turbine blades and designed an unsupervised learning method for intelligent damage identification. This study showed that radiographic testing can visualise and accurately show blade damage, but it suffers from the problems of expensive equipment and harmful effects on the human body. Yiğit et al. [13] used the liquid penetration method to detect aircraft wing components, which can effectively detect impact damage and various scratches on the wings. This study showed that penetration testing can effectively detect microcracks, but penetration takes a long time. The research indicates that microcracks in aero-engine blades are difficult to identify directly through visual inspection or borescope examination. Penetrant testing employs dyeing to enhance microcracks on the blade surface, subsequently enabling visual identification. This constitutes a straightforward inspection method. Consequently, this paper proposes using chromic acid anodisation to treat aircraft engine compressor blades. This treatment renders microcrack regions yellow, significantly enhancing crack visibility. Consequently, rapid and intuitive detection of blade microcracks is achievable under visible and ultraviolet light illumination.

Traditionally, a human being is still required to identify the results after microcracks have been detected using various microcrack detection methods. This work has the following two problems: it has specific requirements for the responsibility, patience and eyesight of the microcrack inspectors, and the inspectors at the site need to be trained for a long time to adapt to this work. Secondly, it is monotonous, repetitive work, and the number of blades is large, which makes it easy for inspectors to become fatigued and bored, affecting the efficiency and accuracy of microcrack detection. In recent years, with the rapid development of computers and the arrival of the big data era, deep learning technology has been rapidly developed and applied. Image recognition has been even more widely studied as a technology analogous to human-eye recognition. AlexNet [14] was proposed in 2012 and won the ImageNet image recognition competition that year. ResNet [15], in 2015, demonstrated a method to solve the profound neural network training challenge through residual connectivity, which became an essential cornerstone in deep learning. The R-CNN series [16,17], YOLO series [18,19], and others proposed afterwards have even brought image recognition to new heights. Therefore, deep learning-based intelligent blade-recognition methods have been extensively researched [20,21]. Beyond blade visual inspection, recent studies have extended intelligent fault diagnosis to aero-engine and rotating-machinery components under limited-data and resource-constrained conditions. Wang et al. [22] proposed a virtual-domain-driven semi-supervised hyperbolic metric network with domain-class adversarial decoupling for aircraft-engine intershaft-bearing fault diagnosis. Wang et al. [23] further developed a neural-dynamics-inspired metric SpikingFormer to achieve energy-efficient mechanical fault diagnosis under insufficient-sample conditions. These studies demonstrate the potential of advanced metric learning and representation-learning strategies for reliable fault diagnosis when labelled data and computational resources are limited. For blade-specific visual inspection, He et al. [24] designed an improved Cascade Mask R-CNN network for engine blade localisation and segmentation, and its accuracy reached 98.81%. Li et al. [25] developed a coarse-to-fine deep learning detection framework for the intelligent identification of microcracks in aero-engine blades, and its accuracy reached 93.5%. Li et al. [26] designed the DDSC-YOLOv5s network for intelligent recognition of engine blade damage collected under endoscopy, and its mAP (mean average precision) value reached 83.8%. Shang et al. [27] used a texture-focused multi-scale feature fusion network and balanced L1 loss to improve Mask R-CNN for intelligent recognition of endoscopic blade damage in situ. Song et al. [28] designed a detection method combining a flexible robotic arm and YOLOv6 to achieve intelligent recognition of tiny defects in small blades. Liu et al. [29] developed an aero-engine blade surface defect detection system based on improved Faster RCNN to address the problems of automatic blade detection difficulty and defect discontinuity on the full image. The system recognised an mAP value of 79.0%. Ma et al. [30] proposed a semantic a priori-guided defect perception network named SPDP-Net. This network realises the detection of surface defects on aero-engine blades in real industrial scenarios, and the detection accuracy reaches 94.9%. The above studies show that deep learning can effectively realise the intelligent recognition of damage. However, the damage they studied can be recognised directly by visual means, and microcracks, which are difficult to detect by visual means alone, are not considered.

Multimodal fusion-based detection methods can utilise rich data to improve the robustness of the model. Park et al. [31] proposed an RGB-D multi-level residual feature fusion network (RDFNet) for indoor semantic segmentation. This network leverages the apparent information from RGB images and the geometric information from depth data through a deep residual learning mechanism to address the key challenge of multi-modal feature fusion. Li et al. [32] developed an end-to-end fusion network architecture (RFN-Nest) for the fusion of infrared and visible images. Infrared images reveal information about an object’s thermal radiation, while visible light images reflect its surface details. By fusing these two images, the accuracy and reliability of object recognition can be improved. Zhang et al. [33] proposed a cross-modal fusion framework (CMX) for unified processing of semantic segmentation tasks involving RGB and multiple modalities. The framework designs a cross-modal feature correction module (CM-FRM) and a feature fusion module (FFM), achieving state-of-the-art performance on nine multi-modal datasets. Du et al. [34] proposed an asymmetric cross-modal network, AsymFormer, for real-time RGB-D semantic segmentation on mobile platforms. The framework adopts a heterogeneous dual-branch architecture, achieving 54.1% mIoU on the NYUv2 dataset. Wu et al. [35] designed a multimodal fusion network, VDFNet. This network not only realises the detection of engine blade damage but also achieves the measurement function by fusing visible light and depth maps. The above studies show that the network model of multimodal fusion can obtain more comprehensive and accurate data dimensions than the unimodal network, which leads to better generalisation ability and robustness.

Existing multimodal fusion methods, such as RDFNet, CMX, and AsymFormer, were mainly developed for RGB-D semantic segmentation and general scene understanding. In contrast, VUFNet is designed for object-level detection of weak, elongated microcracks from paired visible–ultraviolet blade images. Its contribution does not lie in the use of transformer or feature fusion alone, but in their task-oriented integration: CSPSTR combines convolutional local representation with shifted-window self-attention for weak-crack feature extraction; VUFM performs multi-scale visible–ultraviolet fusion through spatial–channel enhancement and gradient-sensitive, normalised weighting; and MFConv captures multi-scale and directional responses associated with elongated crack morphology. These designs distinguish VUFNet from generic multimodal fusion architectures and adapt the network to the specific characteristics of blade microcracks.

In summary, to address the challenge of detecting microcracks on blade surfaces, this paper proposes a novel multi-modal fusion detection network for blade microcracks—VUFNet (visible and ultraviolet multi-modal fusion detection network). It integrates features from visible and ultraviolet images to achieve microcrack detection on blades. It is designed to improve the detection of small microcracks under the visible–ultraviolet imaging conditions evaluated in this study. Experimental validation demonstrates that VUFNet achieves mAP0.5 and mAP0.5:0.95 scores of 96.8% and 85.5%, respectively, with a detection speed of 52.7 fps, enabling efficient identification of blade microcracks. The principal innovations of this paper are as follows:

1. Designing CSPSTR, which combines swin transformer’s sliding window self-attention mechanism with the cross-stage residual structure of C2f to construct a hybrid network with multi-scale perception capabilities, effectively enhancing the model’s microcrack feature extraction ability.
2. Designing VUFM, which achieves adaptive fusion of visible and ultraviolet multi-modal features through a spatial–channel enhancement mechanism and dynamic weight allocation strategy.
3. Designed MFConv, replacing conventional convolutions with multi-branch fusion convolutions. This multi-branch architecture integrates multi-scale, multi-directional convolutional features, enhancing detection accuracy in microcrack target regions.
4. Innovative application of chromic acid anodic oxidation pretreatment combined with multispectral imaging technology. Physicochemical modification induces specific colour reactions in microcrack regions, establishing a visible-to-ultraviolet microcrack dataset.

## 2. VUFNet

Deep learning technology has advanced rapidly in recent years, with numerous high-performance models continually emerging. However, significant challenges persist in detecting microcracks in aero-engine blades, primarily due to the extremely small size of microcracks, their concealed distribution, and their difficulty in detection. This paper proposes VUFNet (visible and ultraviolet multi-modal fusion detection network), a detection network designed for multimodal fusion of visible and ultraviolet images to address this issue. This network simultaneously integrates and learns features from visible and ultraviolet images of the blade surface, thereby achieving more precise microcrack detection.

**Figure 1:**
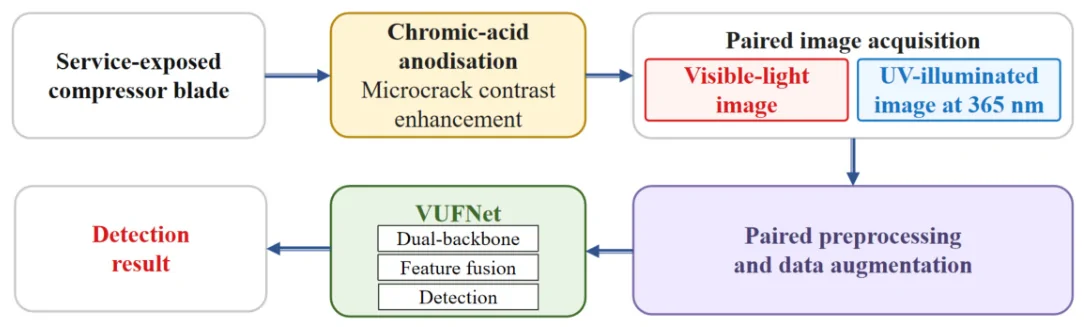
Overall workflow of the proposed visible–ultraviolet microcrack detection method.

Figure 1 presents the overall workflow of the proposed method. After chromic acid anodisation, visible-light and UV-illuminated images are acquired from the same blade region to form a spatially corresponding image pair. The paired images are synchronously preprocessed and then fed into VUFNet to obtain the final microcrack detection results.

**Figure 2:**
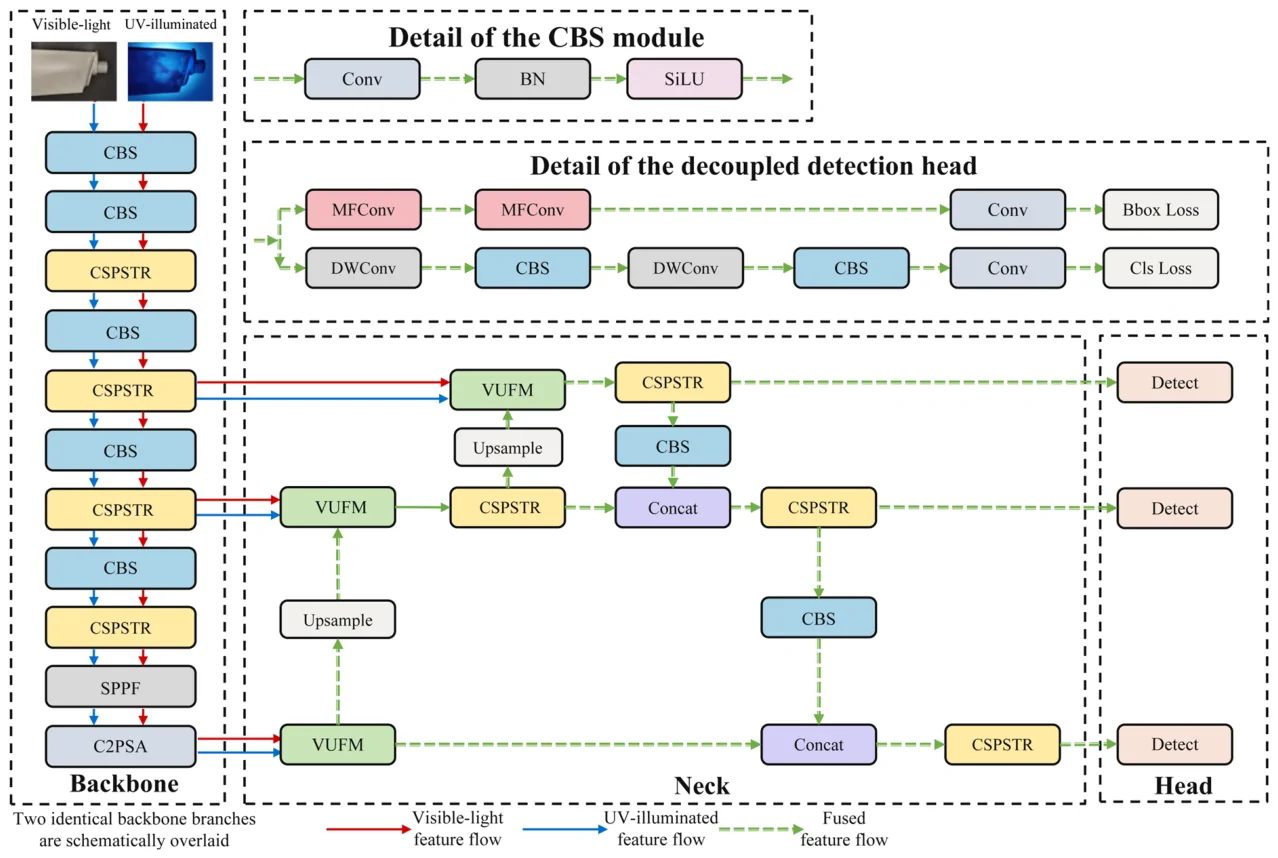
Detailed architecture of the proposed VUFNet.

Figure 2 illustrates the detailed structural design of VUFNet. The network comprises two backbone networks, a neck network and a head network. Both backbone networks employ identical architectures to extract visible and ultraviolet features of blade microcracks, with the extracted features fused within the neck network. The neck network integrates multi-scale fusion features, combining high-semantic-information deep features with high-resolution shallow features. The head network incorporates three detection layers of differing sizes, dedicated to predicting the categories of large, medium, and small targets, alongside bounding box regression. Detection results may be computed as follows:

(1) D=HNet{NNet[BNet(Visible),BNet(Ultraviolet)]},

where D denotes the object detection results, encompassing both classification and localisation of detected objects. Backbone (·), Neck [·], and Head {·}, respectively, refer to the backbone network, neck network, and head network.

For compact presentation, the visible-light and UV-illuminated backbone branches, which have identical architectures, are schematically overlaid. Red and blue arrows represent the visible-light and UV-illuminated feature flows, respectively. Corresponding features from the two branches are fused by VUFM at multiple scales, after which green arrows represent the fused feature flow through the neck and detection head.

CBS, convolution–batch normalisation–SiLU; CSPSTR, cross-stage partial bottleneck with swin transformer; SPPF, spatial pyramid pooling-fast; C2PSA, cross-stage partial block with position-sensitive attention; VUFM, visible–ultraviolet multimodal adaptive fusion module; DWConv, depthwise convolution; MFConv, multi-branch fusion convolution; BN, batch normalisation; SiLU, sigmoid linear unit.

The network builds upon YoloV11 with enhancements: first, CSPSTR was designed, employing window self-attention and a sliding window mechanism to strengthen long-range dependencies, thereby achieving global–local collaborative feature extraction. Subsequently, VUFM was developed to deeply fuse visible light features with the ultraviolet features of microcracks. Finally, MFConv replaces conventional convolutions with multi-scale fusion convolutions. This multi-branch architecture integrates multi-scale, multi-directional convolutional features to enhance detection accuracy for microcrack target regions.

### 2.1. CSPSTR (Cross-Stage Partial Bottleneck with Swin Transformer)

**Figure 3:**
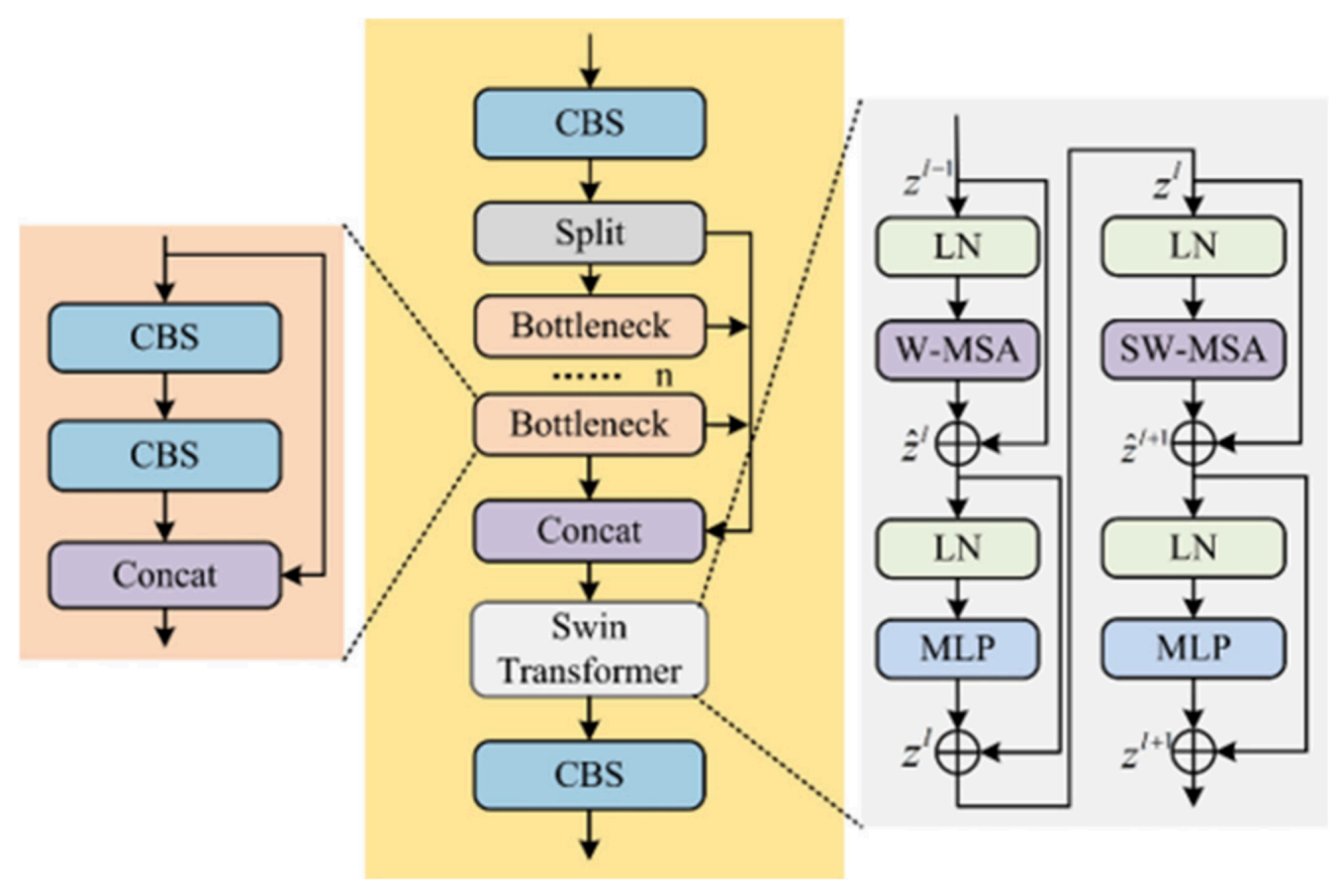
Schematic diagram of CSPSTR.

Figure 3 illustrates the structural design of CSPSTR. Building upon the C2f module, it incorporates swin transformer [36] for enhancement. It extracts and transforms input data features through feature transformation, branch processing, and feature fusion operations to generate more expressive feature maps. The computational process of the CSPSTR module is as follows: first, the input feature map undergoes feature transformation via CBS (comprising a convolutional layer with configurable kernel size/stride, a batch normalisation layer, and an SiLU activation function). Subsequently, the transformed feature map is evenly split into two parts by Split. One part is directly fed backwards, while the other undergoes progressive feature extraction through multiple bottleneck modules (comprising two CBS layers and one Concat layer). Finally, the feature maps from both parts are concatenated along the channel dimension, then undergo further feature extraction via swin transformer and CBS to yield the final feature map. The computational formula for the CSPSTR module is as follows:

(2) $$\begin{array}{l} F_{out}=CBS(\mathrm{SwinTransformer}(Concat(F_{1},F_{2}\cdots ,F_{n})))\\ F_{1}=\frac{1}{2}CBS(F_{in})\\ F_{2}=\mathrm{Bottleneck}(\frac{1}{2}CBS(F_{in}))\\ F_{n}=\prod_{i=1}^{n}{\mathrm{Bottleneck}}_{i}(\frac{1}{2}CBS(F_{in})) \end{array}$$

where $F_{in}$ and $F_{out}$ are the input and output features, respectively. $F_{1},F_{2}\cdots ,F_{n}$ is the feature extracted from the 1st, 2nd, …, and nth branches.

**Figure 4:**
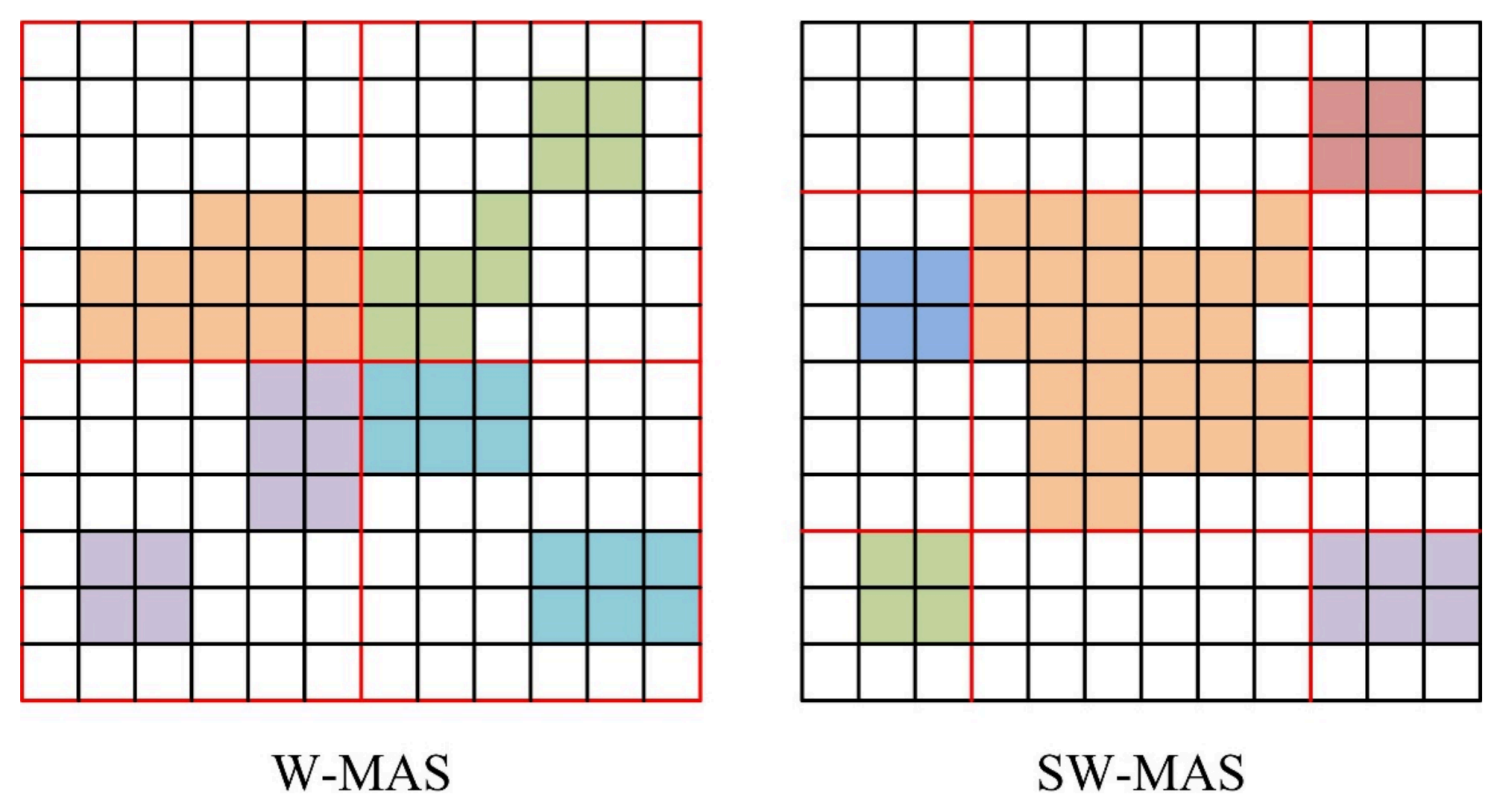
Shift window-based MSA module schematic.

As illustrated in the right-hand portion of Figure 3, the swin transformer combines windowed self-attention (W-MSA) with shifted windowed self-attention (SW-MSA). Figure 4 presents schematic diagrams of W-MSA and SW-MSA. At layer l, a conventional window-partitioning scheme divides the voxel feature map into local windows, enabling self-attention to focus within these regions. This approach enhances computational efficiency and mitigates interference from irrelevant distant pixels. Subsequently, the MLP module employs fully connected layers to adjust and reorganise features processed through the WSA module, enabling the model to utilise better features extracted via the attention mechanism. Finally, features processed through the first residual connection are combined with those extracted via the second residual connection from the MLP module, further increasing the network depth. At layer l + 1, the window division shifts to generate new windows. This is achieved by translating the window positions along the X and Y axes by the same number of steps (here, 3). Consequently, the self-attention computations within the new windows extend beyond the boundaries of the preceding layer’s windows, thereby establishing connections between different windows. The swin transformer’s computational formula is as follows:

(3) $$\begin{array}{l} {\hat{z}}^{l}=W-MSA(\mathrm{LN}(z^{l-1}))+z^{l-1}\\ z^{l-1}=\mathrm{MLP}(LN({\hat{z}}^{l}))+{\hat{z}}^{l}\\ {\hat{z}}^{l+1}=\mathrm{SW}-MSA(LN(z^{l-1}))+z^{l-1}\\ z^{l+1}=\mathrm{MLP}(LN({\hat{z}}_{3}^{l+1}))+{\hat{z}}^{l+1} \end{array}$$

where ${\hat{z}}^{l}$ and $z^{l}$ denote the (S)W-MSA module output features and MLP module output features of the lth block, respectively. W-MSA and SW-MSA denote window-based multi-head self-attention using regular and shifted window-partitioning configurations, respectively.

### 2.2. VUFM (Visible–Ultraviolet Multi-Modal Adaptive Fusion Module)

**Figure 5:**
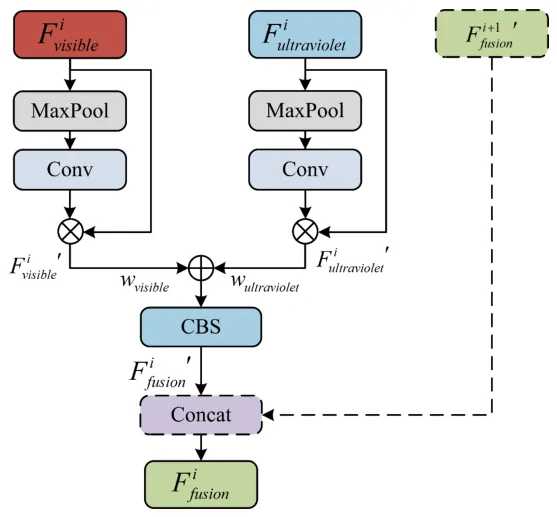
Schematic diagram of VUFM.

Figure 5 illustrates the structural design of VUFM. Visible and ultraviolet features are first enhanced through a max-pooling layer to improve the model’s local feature extraction capability, followed by channel dimension adjustment via convolutional layers. Subsequently, the processed features undergo element-wise multiplication with the original input through skip connections, forming a spatial–channel adaptively enhanced feature mechanism. The enhanced multimodal features undergo weighted fusion via a gradient-sensitive weight allocation module, which dynamically computes normalised fusion coefficients from the confidence scores derived from the gradient magnitudes of the visible and ultraviolet feature maps. As defined in Equations (5)–(7), the coefficients satisfy 0 ≤ $w_{visible}$, $w_{ultraviolet}$ ≤ 1 and $w_{visible}$ + $w_{ultraviolet}$ = 1, and are not independently optimised as free learnable parameters. This normalisation limits unbounded weight drift and mitigates the risk of persistent collapse toward a single modality, while still allowing one modality to receive a larger coefficient for an individual sample when it exhibits a stronger microcrack-edge response. The weighted features are subsequently processed through the CBS module for further feature extraction. When upper-layer fusion features are present, a feature superposition strategy is employed to achieve cross-level information integration, ultimately outputting fused features that combine multi-scale perception with multimodal synergy. The complete formula for the fusion process is as follows:

(4) Fvisiblei=′Fvisiblei×Conv(MaxPool(Fvisiblei))Fultravioleti=′Fultravioleti×Conv(MaxPool(Fultravioleti))Ffusioni=′CBS(wvisible⋅Fvisiblei+′wultraviolet⋅Fultravioleti)′Ffusioni=Concat(Ffusioni,′Ffusioni+1)′

Gradient sensitivity weights dynamically assign fusion weights to enhance the characterisation of microcracked regions by quantifying the gradient magnitude of the feature map. The weight allocation process of this mechanism is directly related to the gradient response of the microcrack edges, and the fusion weights can be dynamically adjusted without introducing additional learning parameters, which improves the feature fusion effect while maintaining the computational efficiency. Specifically, given the visible features $F_{visible}\in {\mathbb{R}}^{C\times H\times W}$ and ultraviolet features $F_{ultraviolet}\in {\mathbb{R}}^{C\times H\times W}$, the gradient magnitude matrix of each mode is first calculated:

(5) $$\begin{array}{l} M_{visible}=\sqrt{{\left(S_{x}\times F_{visible}\right)}^{2}+{\left(S_{y}\times F_{visible}\right)}^{2}}\\ M_{ultraviolet}=\sqrt{{\left(S_{x}\times F_{ultraviolet}\right)}^{2}+{\left(S_{y}\times F_{ultraviolet}\right)}^{2}} \end{array}$$

where $S_{x}$ and $S_{y}$ are the Sobel horizontal and vertical operators, respectively. The modal level confidence is obtained by global average pooling:

(6) $$\begin{array}{l} {\mathrm{Conf}}_{visible}=\frac{1}{CHW}\sum_{c,i,j}M_{visible}^{c,i,j},\\ {\mathrm{Conf}}_{ultraviolet}=\frac{1}{CHW}\sum_{c,i,j}M_{ultraviolet}^{c,i,j} \end{array}$$

The final normalised dynamic weights are generated:

(7) $$w_{visible}=\frac{{\mathrm{Conf}}_{visible}}{{\mathrm{Conf}}_{visible}+{\mathrm{Conf}}_{ultraviolet}},w_{ultraviolet}=1-w_{visible}$$

### 2.3. MFConv (Multi-Branch Fusion Convolution)

The detection module comprises regression and classification branches. Since this study focuses on single-category microcracks, enhancing the regression branch’s performance to detect microcrack regions precisely becomes paramount. Traditional convolutions have limitations such as a singular receptive field and fixed sampling patterns, making it challenging to balance local details with global contextual information. Furthermore, they lack robustness against target scale variations and morphological deformations. To address these issues, this paper draws inspiration from MBRConv (multi-branch re-param convolution) [37] to design MFConv as a replacement for the original convolutional structure. By employing a multi-branch architecture to fuse multi-scale, multi-directional convolutional features, MFConv enhances the detection accuracy of microcrack target regions.

**Figure 6:**
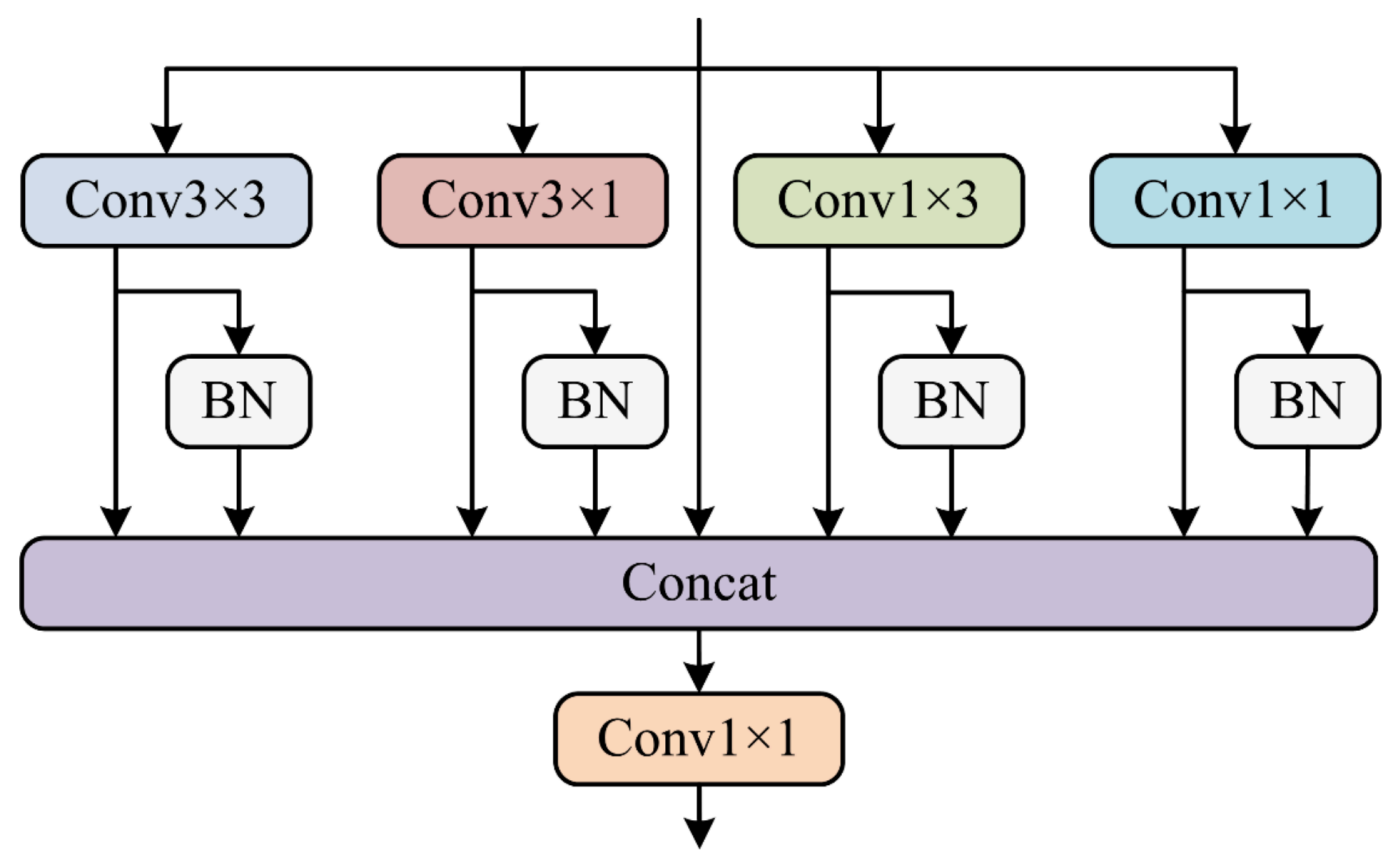
Schematic diagram of MFConv.

Figure 6 illustrates the structural design of MFConv. MFConv comprises five parallel branches: 1 × 1 convolution, 3 × 3 convolution, 1 × 3 convolution, 3 × 1 convolution, and identity mapping. Each convolution branch is followed by identity mapping and batch normalisation to enhance training stability. The outputs from each branch are concatenated along the channel dimension and then fused through a 1 × 1 convolution to generate the final features. This architecture explicitly decouples and synergistically models multi-scale and multi-directional information: the 1 × 1 branch handles channel transformation and semantic reorganisation, reducing computational complexity while enhancing feature expressiveness; the 3 × 3 branch captures local textures and multi-scale crack morphology, while the 1 × 3 and 3 × 1 branches enhance anisotropic structural responses in horizontal and vertical directions, respectively, improving sensitivity to elongated cracks. The identity mapping preserves original features and mitigates gradient vanishing. Through multi-branch feature fusion, MFConv effectively integrates detailed edge and global contour information in scenarios with low contrast, elongated cracks, or significant noise interference, substantially enhancing the model’s feature representation capability and small object detection accuracy. The calculation formula for MFConv is as follows:

(8) $$\begin{array}{l} F_{output}=\mathrm{Conv}1\times 1(Concat(F_{1},\mathrm{BN}(F_{1}),\\ F_{2},\mathrm{BN}(F_{2}),F_{3},\mathrm{BN}(F_{3}),F_{4},\mathrm{BN}(F_{4}),F_{input}))\\ F_{1}=\mathrm{Conv}3\times 3(F_{input})\\ F_{2}=\mathrm{Conv}3\times 1(F_{input})\\ F_{3}=\mathrm{Conv}1\times 3(F_{input})\\ F_{4}=\mathrm{Conv}1\times 1(F_{input}) \end{array}$$

### 2.4. VUFNet-Data and VUFNet-Decision

Depending on the fusion stage, multimodal fusion detection can be categorised into data-level fusion, feature-level fusion, and decision-level fusion. Beyond designing the feature-level fusion model VUFNet, this paper also proposes VUFNet-data and VUFNet-decision to comprehensively investigate the impact of different fusion stages on multimodal model performance. VUFNet-data implements data-level fusion by integrating visible and ultraviolet data, then employing a neural network to extract microcrack features. VUFNet-decision implements result-level fusion by processing visible and ultraviolet data through separate networks for detection. Finally, the microcrack detection results from both networks are fused using non-maximum suppression.

## 3. Experimental Setup

### 3.1. Blade Treatment

**Figure 7:**
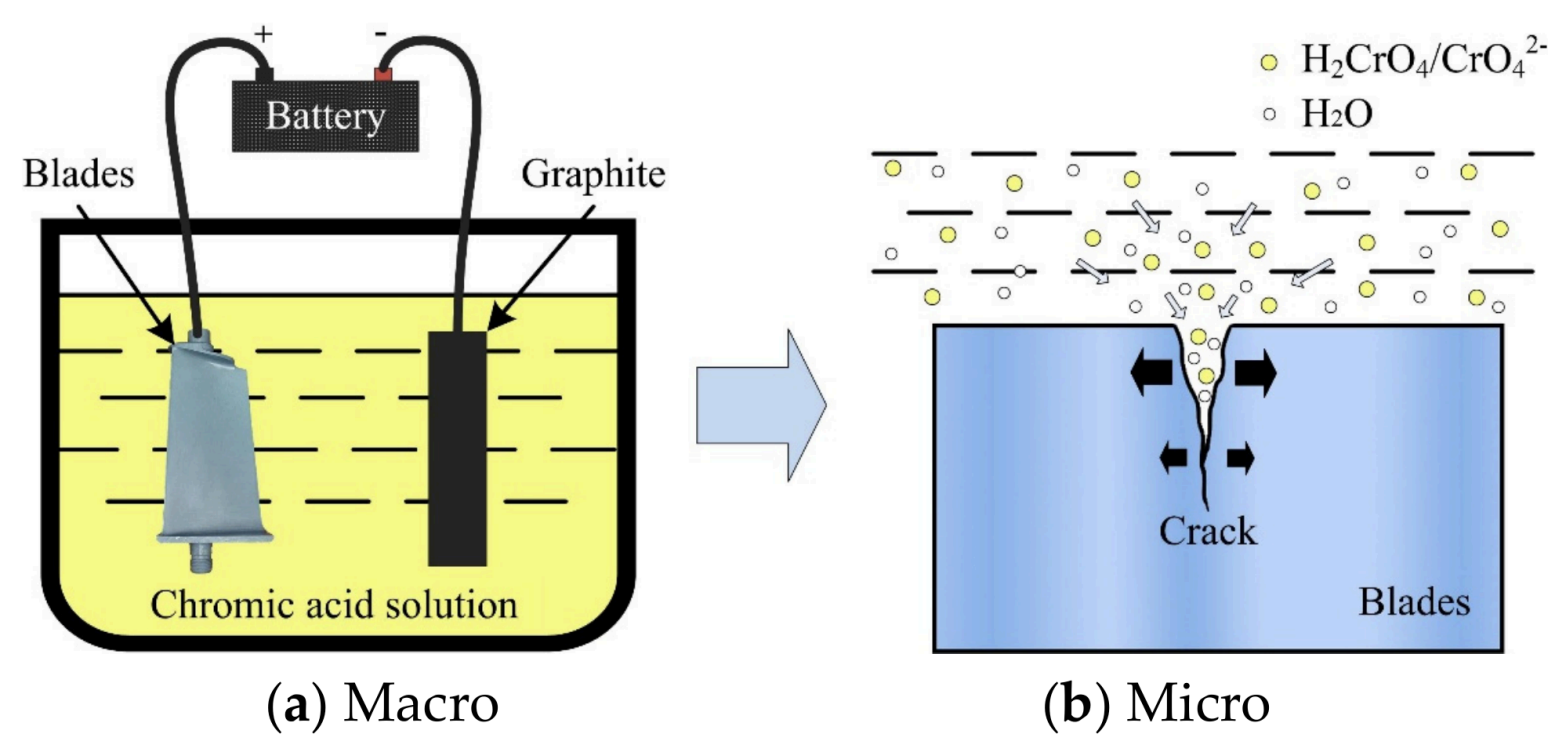
Schematic diagram of chromic acid anodizing.

Microcracks in aircraft engine blades are characterised by their small size and hidden locations, making them invisible to the naked eye. To address this issue, this paper proposes the use of chromic acid anodisation technology to treat the blades, causing the microcrack areas to appear yellow, thereby enhancing the detectability of the microcracks [38]. Figure 7 illustrates the principle of chromic acid anodisation. (a) The experiment was conducted in a chromic acid electrolyte solution, with the main components being H_2_CrO_4_/CrO_4_^2−^, at a concentration of 80 ± 5 g/L. In the experiment, the metal blade serves as the anode connected to the positive terminal of the power source, while graphite acts as the cathode connected to the negative terminal. Under these conditions, a direct current of 220 V is applied for 30 min, resulting in the formation of a dense oxide layer on the surface of the metal blade. All blades in this study were treated using the same anodisation parameters (chromic acid concentration of 80 ± 5 g/L, voltage of 220 V, and treatment duration of 30 min) to ensure consistency of the collected dataset. These parameters were selected based on preliminary trials to achieve sufficient chromic acid penetration and contrast enhancement of microcrack regions. (b) During the chromic acid anodisation process, the surface of the blade is subjected to anodic corrosion, causing previously covered or closed microcracks to open, thereby revealing their inherent defects. Due to the high permeability and low viscosity of the chromic acid solution, it can rapidly penetrate the microcracks. After washing the blade with water and allowing it to stand for 1 h, the chromic acid within the microcracks seeps out, causing them to appear yellow.

**Figure 8:**
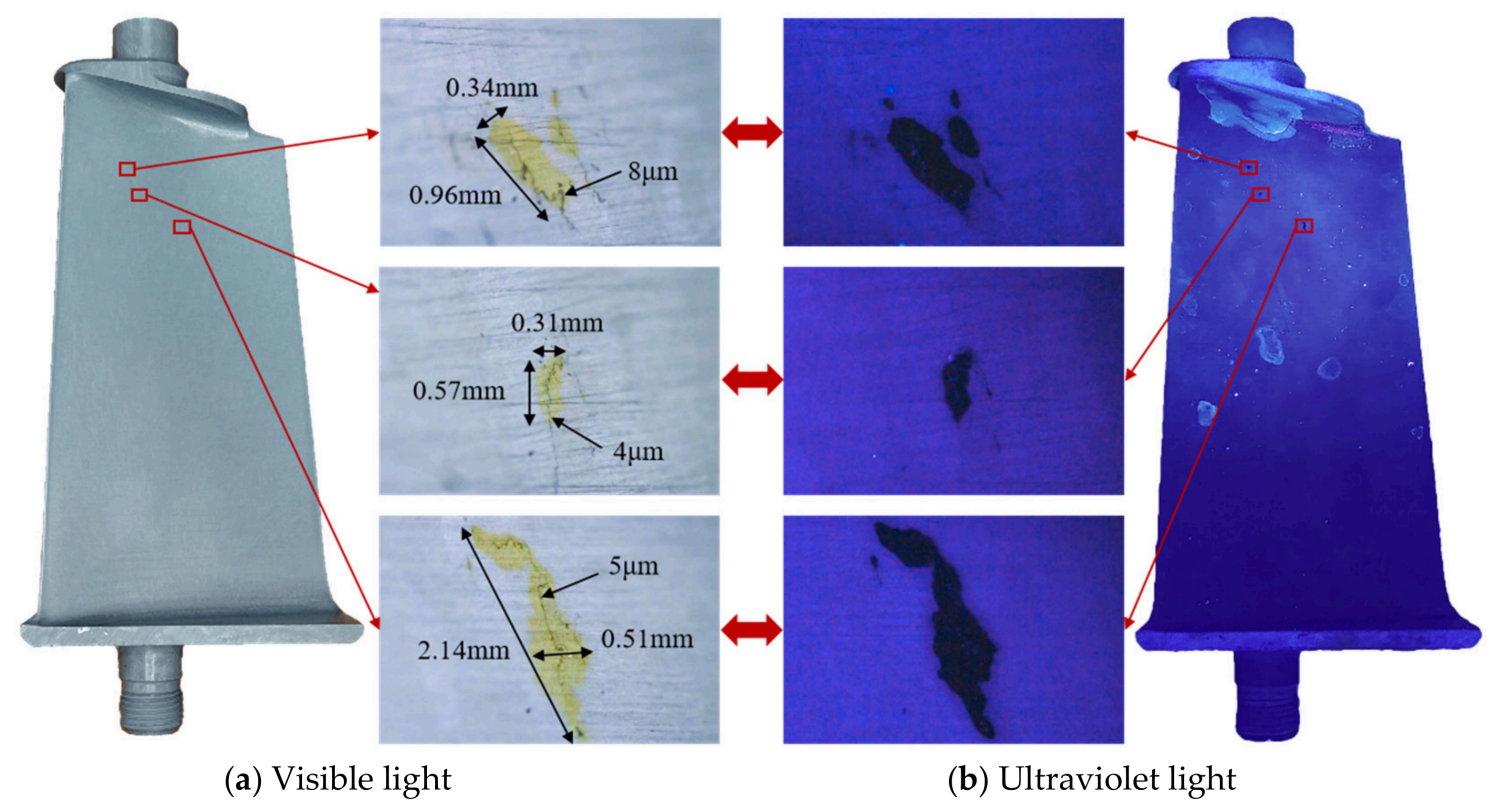
Blade microcrack images. (**a**) Visible light observation of microcracks on the blade surface; (**b**) Ultraviolet light fluorescence detection results of the corresponding microcracks.

Figure 8 shows the image of the resultant microcracks captured after chromic acid anodisation of the blade. The image in 8(a) is captured under visible light irradiation, and faint yellow dots can be detected from the global image of the blade. After high magnification, it can be seen that the length of the cracks is at the millimetre level, but the width is only at the micron level. Due to chromic acid exudation, a large area of yellow colouration appears around the crack, and this yellow area is the same as the crack in the length direction. However, it is expanded in the width direction. Taking the bottom enlargement as an example, the width of the crack is 5 μm, and the widest part of the yellow colour reaches 0.51 mm, which is an increase of 102 times. This indicates that the chromic acid anodising treatment can significantly enhance the characteristics of blade microcracks. The image in 8(b) is acquired under 365 nm ultraviolet light irradiation. Since chromic acid has the property of absorbing ultraviolet, obvious black spots can be found in the global image of the blade. A black area can be found after high magnification. By comparison, this black area is consistent with the yellow area captured under fluorescent light, and its contrast with the surrounding area is significantly improved.

### 3.2. Constructing a Microcrack Dataset

This experiment collects a specific type of service engine compressor blades from the factory, which have been damaged by the complex service environment. Using a high-precision industrial camera, we took photos of the blades after the chromic acid anodising treatment. The camera models are the MK1628M lens and the A7500CG20 camera were manufactured by Zhejiang Huaray Technology Co., Ltd., Hangzhou, China. Selecting different shooting distances, different shooting angles, and different light source irradiation to collect data improves the diversity of data so that the final model has a stronger recognition ability. To ensure data consistency, we use the exact location to take photos under visible light and ultraviolet light irradiation, and finally collect a total of 2000 pairs of data, which are divided into a training set, validation set and test set according to the ratio of 8:1:1.The dataset contains 2000 spatially registered visible–ultraviolet image pairs acquired from 200 physical blades belonging to a single compressor-blade type. To prevent information leakage caused by multiple views of the same specimen, dataset partitioning was performed at the physical-blade level rather than at the individual-image level. All visible and ultraviolet image pairs, shooting angles, and shooting distances associated with the same physical blade were assigned exclusively to one subset. The resulting training, validation, and test subsets contained 160, 20, and 20 blades.

**Figure 9:**
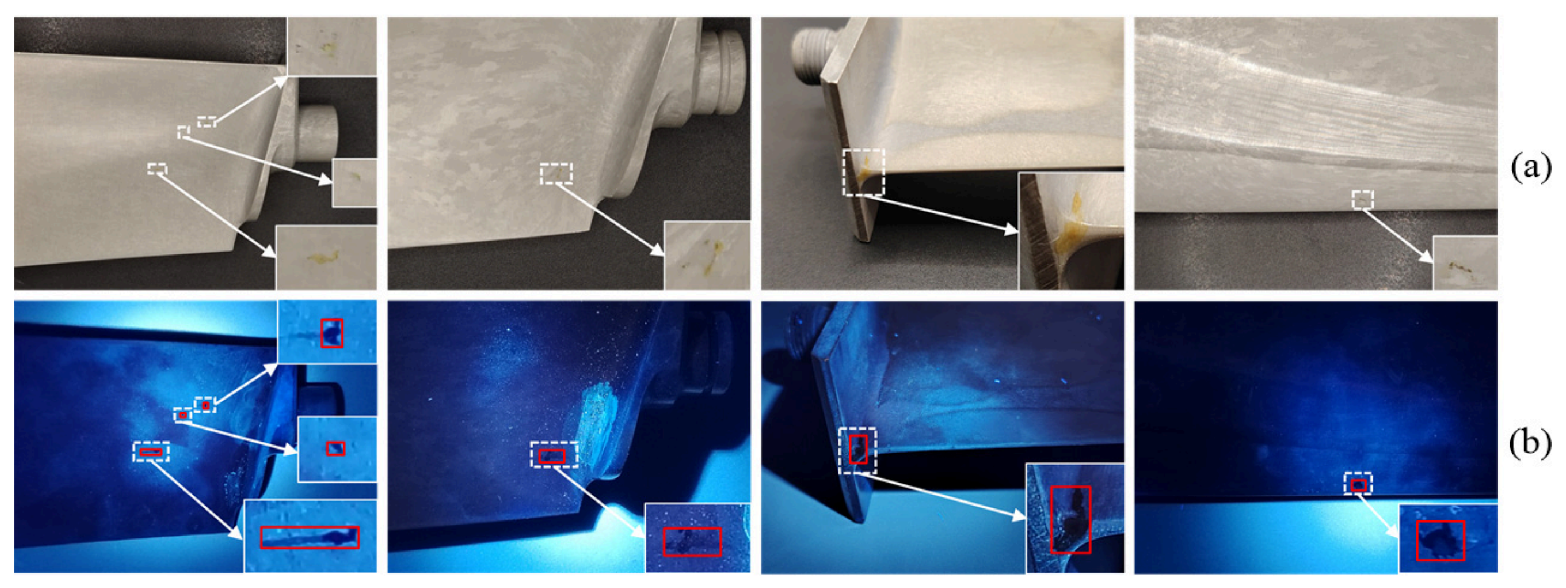
Partial blade microcrack data. (**a**) Visible light photographs showing microcrack features on blade surfaces; (**b**) Ultraviolet fluorescence images revealing the corresponding microcrack defects.

Figure 9 shows some of the data, where (a) is the image collected under visible light irradiation. The microcracks in this type of data are light in colour and difficult to distinguish, but the background is clean and free of interfering targets. The image in (b) is acquired under 365 nm ultraviolet light irradiation. The microcrack features of this type of data are more evident to distinguish, but the background is cluttered, which makes the model easy to misjudge. The data corresponding to the top and bottom of the figure are taken from different light sources at the exact location and are regarded as a group. The image size used in this paper is 640 × 480. This experiment utilises “Labelimg” to label the data manually. The type of labelling is “microcrack”, and the labelled area is based on the image captured under ultraviolet. The red box in Figure 9b shows the labelled area.

**Figure 10:**
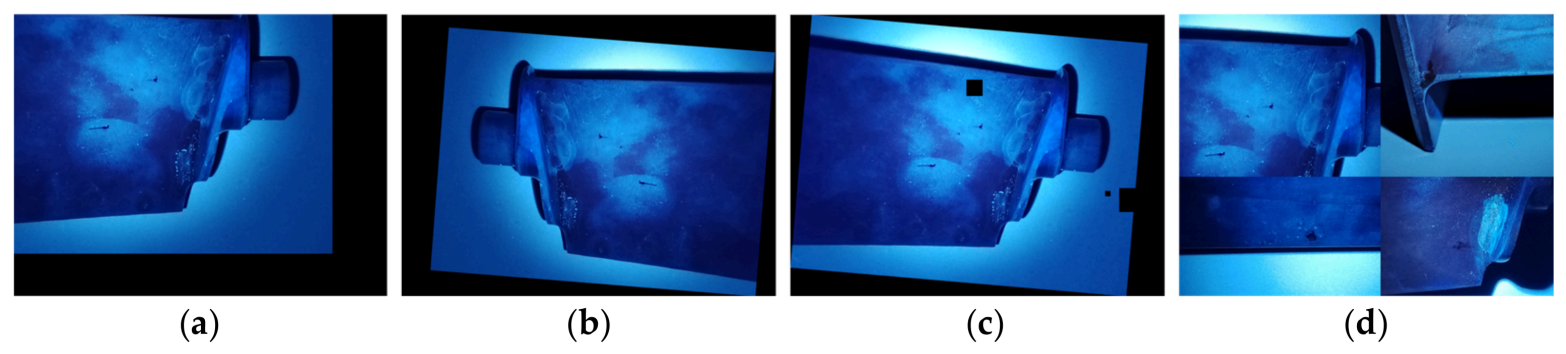
Data augmentation effect diagrams: (**a**) panning; (**b**) combination of panning, flipping and rotating; (**c**) combination of panning, rotating, cutout and changing brightness; (**d**) mosaic.

Rich data are the basis of deep learning. We perform data augmentation based on the original image for better training results. Data augmentation methods include panning, flipping, rotating, cutout, changing brightness, mosaic, etc. These data augmentation methods can be applied to the images individually or in combination. Finally, we enhance the training set from 1600 pairs to 8000 pairs. Some of the data enhancement results are shown in Figure 10.

### 3.3. Evaluation Indicators

We employ mAP_0.5_, mAP_0.5:0.95_, and speed as evaluation metrics to detect microcracks in blades. The following parameters feature in the formulas for specific evaluation metrics: TP (true positives), FP (false positives), FN (false negatives), and TN (true negatives). IoU (intersection over union) denotes the intersection ratio of the concatenation between the bounding box and the actual box. Based on these parameters, standard evaluation metrics for object detection models can be calculated: precision, recall, AP (average precision), and mAP (mean average precision). mAP_0.5_ denotes the average precision when the detection model’s IoU is set to 0.5, while mAP_0.5:0.95_ represents the average precision when the detection model’s IoU is set between 0.5 and 0.95 (with 0.05-interval values). Speed denotes the pure network inference throughput, measured in frames per second (fps). Specifically, it is calculated based only on the forward-pass time of the trained network after the input tensors have been loaded onto the GPU. Image acquisition, disk I/O, image preprocessing, CPU–GPU data transfer, and post-processing operations, such as confidence filtering and non-maximum suppression, are not included. For VUFNet, one input sample consists of one spatially registered visible–ultraviolet image pair. Therefore, the reported speed is used to compare the computational efficiency of different network models under the same experimental platform, rather than to represent the end-to-end throughput of a complete inspection system. The calculation formula is

(9) $$\begin{array}{l} Precision=\frac{TP}{TP+FP}\\ Recall=\frac{TP}{TP+FN}\\ AP=\int_{0}^{1}Precision(\;Recall\;)d(\;Recall\;)\\ mAP=\frac{1}{N}\sum_{i=1}^{N}AP_{i} \end{array}$$

### 3.4. Model Training

In this paper, we use Windows 10 operating system, the compiler is Python 3.10.14, Pytorch 2.1.1, CUDA 11.8. The optimizer-related hyperparameters were adopted from the standard training configuration of the YOLO-based implementation and were not specifically tuned on the test set. All models are trained, validated, and reasoned on NVIDIA RTX3080 (16 GB), and the hyperparameters for training are shown in Table 1.

**Figure 11:**
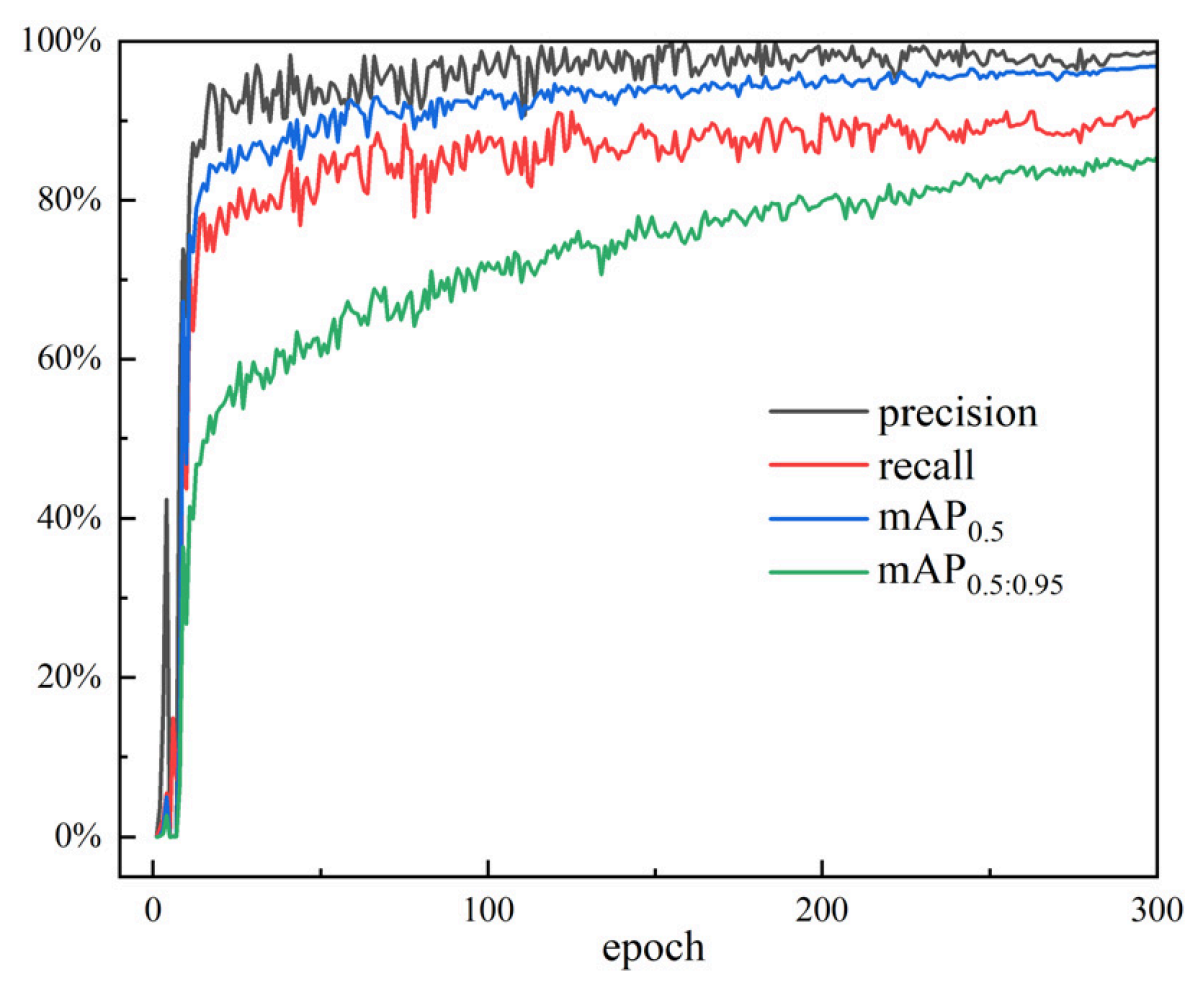
Evaluation indicator change curve chart.

Figure 11 illustrates the progression of four evaluation metrics—precision, recall, mAP_0.5_, and mAP_0.5:0.95_—throughout the training process. The graph demonstrates that as training iterations increase, all metrics exhibit a steady upward trend before stabilising. This phenomenon indicates that the model training process is efficient and stable, capable of thoroughly learning and extracting data features with good convergence, and without overfitting. Specifically, the model performs excellently on the validation set: precision reaches 98.7%, indicating that the proportion of samples predicted as positive that are actually positive is exceptionally high; recall reaches 91.3%, demonstrating that the model can effectively identify most positive samples; mAP_0.5_ reached 96.8%, reflecting the model’s outstanding overall detection performance at an IoU threshold of 0.5; mAP_0.5:0.95_ achieved 85.5%, further demonstrating the model’s sustained high detection accuracy across different IoU thresholds. These results indicate stable convergence and strong within-domain performance under the blade type, surface-treatment protocol, and imaging conditions used in this study.

## 4. Experimental Results and Analysis

### 4.1. Comparative Experiment

**Figure 12:**
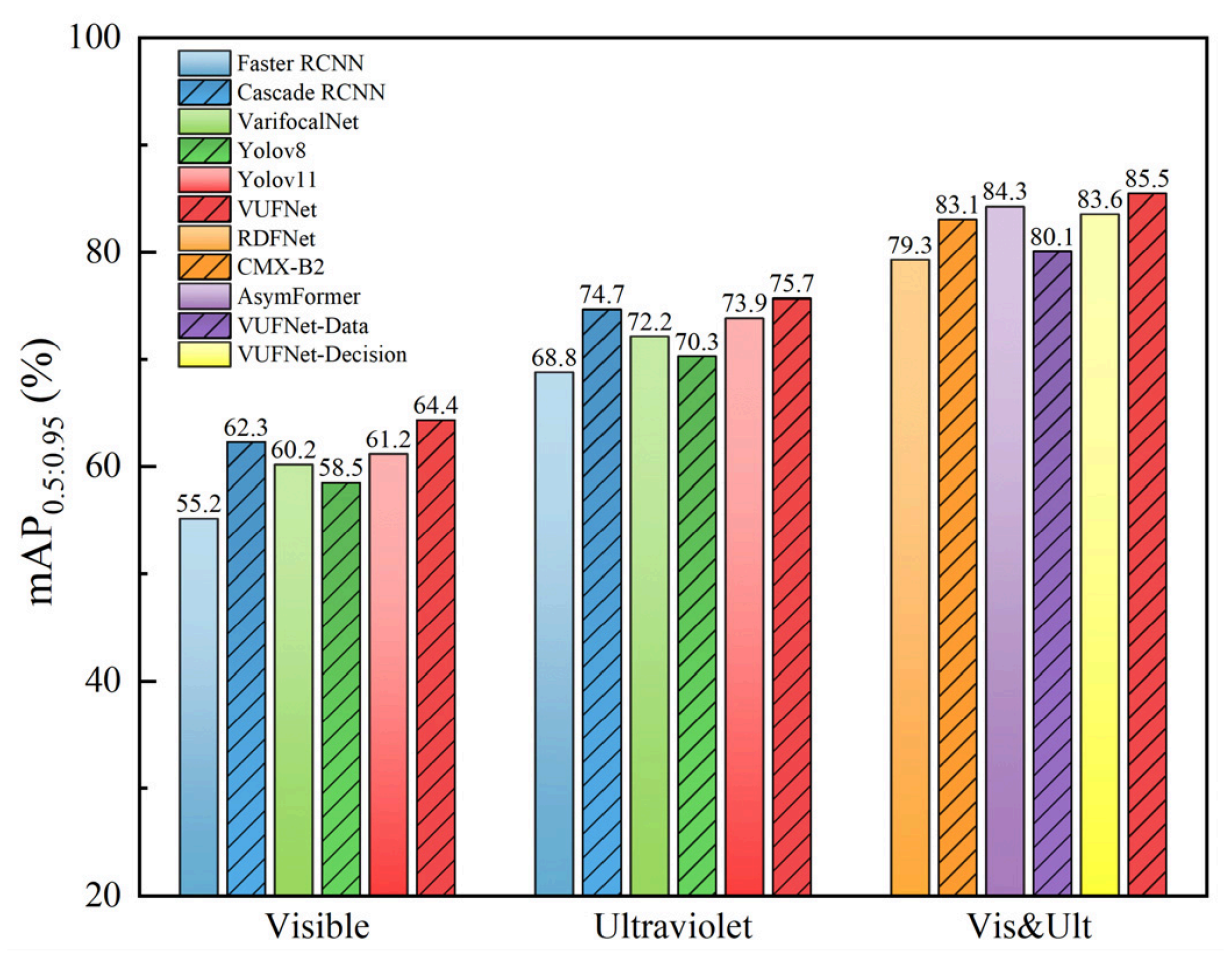
Bar chart of mAP_0.5:0.95_ for each model.

To validate the effectiveness of the proposed microcrack detection method for aero-engine blades, the comparative methods were divided into two categories. The first category included representative object detection networks, namely Faster R-CNN [16], Cascade R-CNN [17], VarifocalNet [39], YOLOv8 [40], and YOLOv11 [41]. The second category included representative multimodal fusion architectures, namely RDFNet [31], CMX-B2 [33], and AsymFormer [34], as well as the proposed VUFNet-Data and VUFNet-Decision variants. It should be noted that RDFNet, CMX-B2, and AsymFormer were originally developed for semantic segmentation rather than object detection; therefore, they were included primarily as references for comparing multimodal feature-fusion strategies rather than as strictly equivalent object-detection baselines. Quantitative evaluations are detailed in Table 2, with the mAP_0.5:0.95_ results illustrated in Figure 12.

Table 2 presents the comparison results between this paper’s model and other models. Visible modality: VUFNet achieved the best performance, attaining mAP_0.5_ of 78.8% and mAP_0.5:0.95_ of 64.4%; whereas Faster RCNN performed the worst, with an mAP_0.5:0.95_ of merely 55.2%. Regarding speed, YoloV11 proved the fastest (87.3 fps), while Cascade RCNN was the slowest (12.1 fps). VUFNet, whilst maintaining the highest accuracy, achieved a significantly quicker speed (52.7 fps) compared to Cascade RCNN and VarifocalNet (38.6 fps), establishing itself as the optimal choice for visible light tasks. It should be emphasised that the fps values reported in Table 2 represent pure network inference throughput under the same RTX 3080 platform and exclude preprocessing, data transfer, and post-processing latency. Therefore, these results are primarily intended for relative computational-efficiency comparison between the evaluated models.

Ultraviolet Modality: All models demonstrated higher detection accuracy under ultraviolet light than in the visible light modality, confirming ultraviolet light’s superior detectability of microcracks. For instance, Faster RCNN’s mAP_0.5:0.95_ improved from 55.2% to 68.8%, while Cascade RCNN surged from 62.3% to 74.7%. VUFNet maintained its lead with an mAP_0.5:0.95_ of 75.7%. Inference speed remained consistent across single modalities.

Visible and Ultraviolet Fusion Modality (Vis&Ult): Under the unified detection evaluation protocol used in this study, VUFNet achieved mAP_0.5_ and mAP_0.5:0.95_ values of 96.8% and 85.5%, respectively. CMX-B2 and AsymFormer achieved mAP_0.5:0.95_ values of 83.1% and 84.3%, respectively. Because CMX-B2 and AsymFormer were originally designed for semantic segmentation, these results should be interpreted as references for evaluating different multimodal fusion strategies under the present dataset and experimental settings, rather than as a strict ranking of native multimodal object-detection networks. VUFNet also maintained a detection speed of 52.7 fps, demonstrating a favourable balance between detection accuracy and computational efficiency under the evaluated conditions.

In summary, microcrack characteristics were more pronounced under ultraviolet illumination than under visible illumination, while visible–ultraviolet fusion further improved detection performance. Among the methods evaluated on the present dataset, VUFNet achieved the highest detection accuracy while maintaining a processing speed of 52.7 fps.

Figure 12 presents the histograms of mAP_0.5:0.95_ for each model. The figure indicates that performance follows the order: fusion modality (Vis&Ult) > ultraviolet modality (Ultraviolet) > visible modality (Visible). Among the three sets of results, VUFNet consistently achieves the highest mAP_0.5:0.95_. Faster RCNN’s mAP_0.5:0.95_ in Visible is 13.6% lower than in Ultraviolet. YoloV11’s mAP_0.5:0.95_ in Visible is 12.7% lower than in Ultraviolet. This indicates that the microcrack features under ultraviolet illumination are superior to those in visible light images. Within VUFNet, the mAP_0.5:0.95_ achieved through Vis&Ult fusion detection surpassed visible-light detection by 32.8% and ultraviolet detection by 12.9%. This demonstrates that Vis&Ult fusion detection effectively captures features from visible and ultraviolet images, yielding superior performance. Under Vis&Ult fusion detection, VUFNet’s mAP_0.5:0.95_ outperformed the independently established RDFNet, CMX-B2, and AsymFormer methods by 7.8%, 2.9%, and 1.4%, respectively, demonstrating the competitiveness of VUFNet relative to external multimodal approaches on the present dataset. VUFNet also outperformed the internally constructed VUFNet-Data and VUFNet-Decision variants by 6.7% and 2.3%, respectively. The latter comparison is interpreted only as a controlled within-framework analysis, indicating that feature-level fusion is more effective than the implemented data-level and decision-level fusion strategies under the same backbone, dataset, and experimental settings.

The consistently higher mAP of VUFNet can be explained by different factors in the single-modality and fusion settings. In the visible-only and ultraviolet-only cases, CSPSTR combines local convolutional representations with shifted-window self-attention, thereby improving the extraction of weak, elongated, and spatially sparse microcrack features. Meanwhile, MFConv aggregates multi-scale and multi-directional responses, which is beneficial for localising microcracks with different sizes and orientations. In the visible–ultraviolet fusion case, VUFM further performs adaptive spatial–channel enhancement and dynamic weighting of the paired features. This enables the model to exploit the relatively clean background information in visible-light images and the stronger microcrack contrast in ultraviolet images, while reducing modality-specific interference. These explanations are also supported by the ablation results, in which removing VUFM, CSPSTR, and MFConv reduced mAP@0.5:0.95 by 2.9, 1.4, and 0.7 percentage points, respectively. Therefore, the superior performance of VUFNet is mainly attributed to the complementary visible–ultraviolet information and the synergistic contributions of the three task-oriented modules.

**Figure 13:**
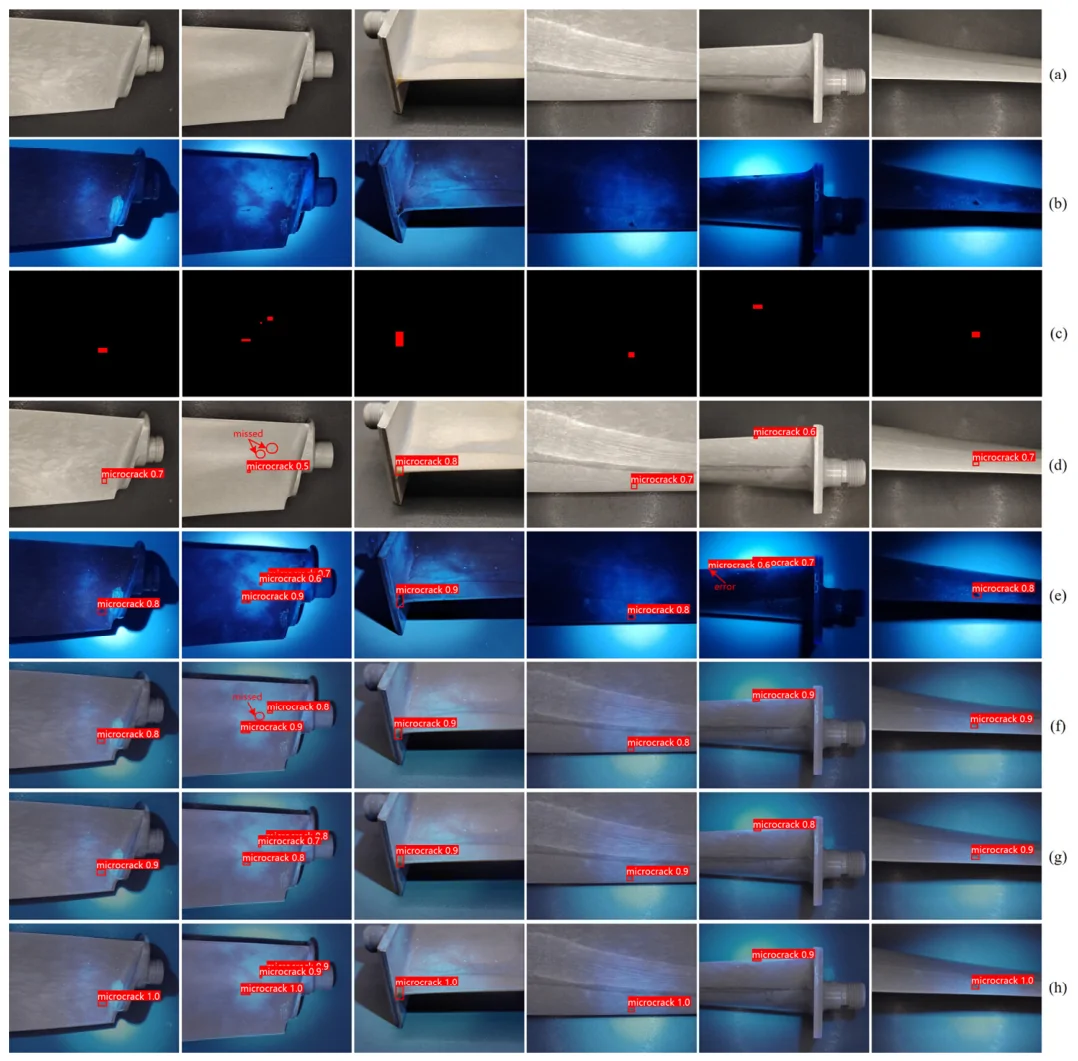
Comparison of microcrack detection results: (**a**) Visible, (**b**) Ultraviolet, (**c**) Ground Truth, (**d**) VUFNet-Visible, (**e**) VUFNet-Ultraviolet, (**f**) VUFNet-Data-Vis&Ult, (**g**) VUFNet-Decision-Vis&Ult, (**h**) VUFNet-Vis&Ult.

Figure 13 presents a comparison of microcrack detection results: (a) shows the visible light image, (b) the ultraviolet image, (c) the ground truth where red boxes denote manually annotated microcrack locations, (d) the detection results from VUFNet using visible light, (e) the detection results from VUFNet using ultraviolet light, and (f) the detection results from VUFNet using a fusion of visible and ultraviolet light. In (g), the results are presented from VUFNet-Data under visible-light and ultraviolet fusion detection, while (h) shows the results from VUFNet-Decision under the duplicate fusion detection. The figures reveal that in (d), two smaller microcracks were missed, and the confidence levels for identifying other microcracks were relatively low. This indicates that identifying microcracks in visible light images is highly challenging, resulting in suboptimal detection performance from the trained model. In (e), one false positive target is present, yet the detection performance is markedly superior to that in (d). This demonstrates that ultraviolet images enhance microcrack features significantly, substantially improving detection efficacy. However, the chaotic background in these images can induce false positives. In (f), the model detects all microcracks with high confidence. This indicates that the fusion detection method combines microcrack features from visible and ultraviolet images, effectively addressing the limitations of single-modality detection and yielding superior overall performance. Missed detections are exhibited in (g), while (h) displays lower confidence than (f). These findings demonstrate that VUFNet surpasses VUFNet-Data and VUFNet-Decision in detection capability. VUFNet represents a high-precision, high-speed microcrack detection method with significant engineering application value.

### 4.2. Ablation Experiment

**Figure 14:**
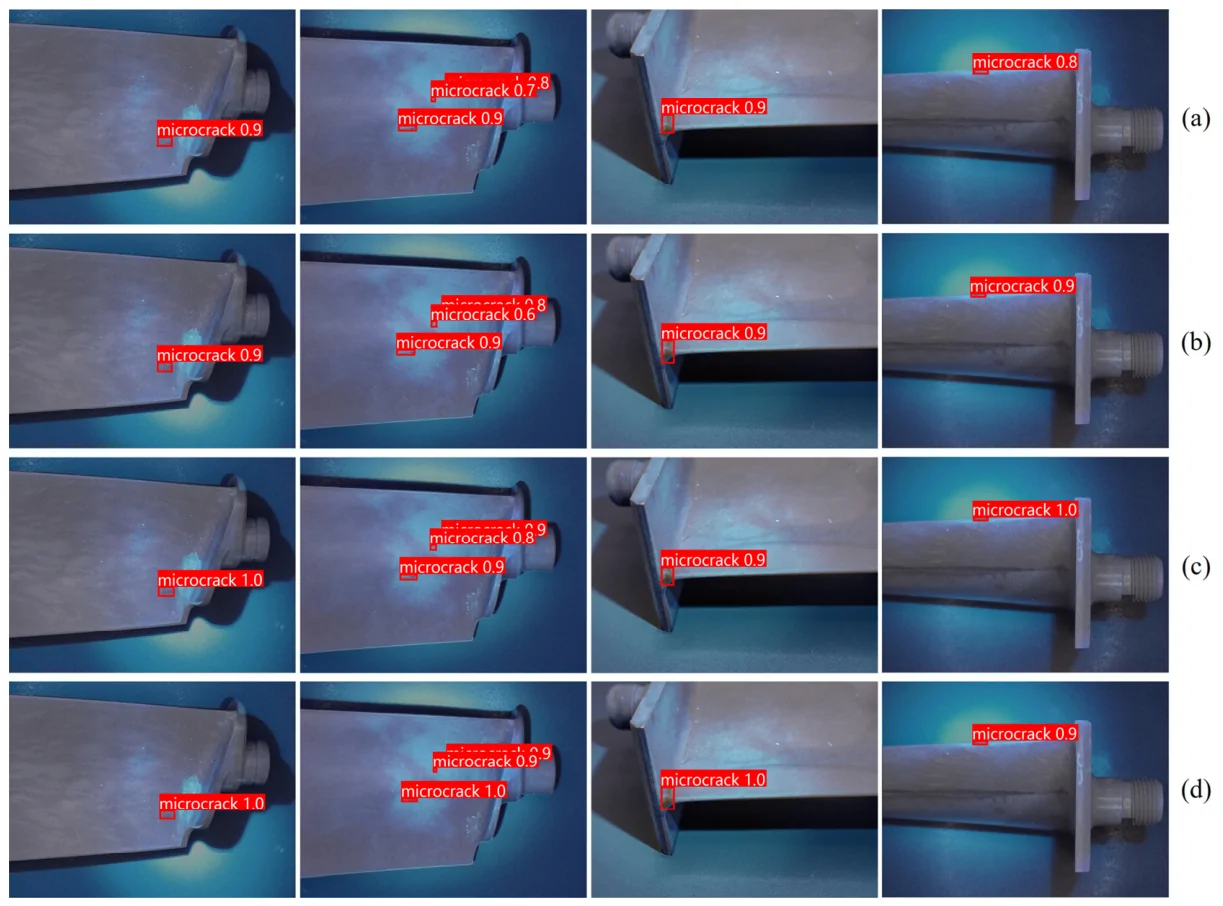
Microcrack detection results: (**a**) VUFNet-without CSPSTR, (**b**) VUFNet-without VUFM, (**c**) VUFNet-without MFConv, (**d**) VUFNet.

This section conducts ablation experiments to investigate the contributions of VUFM, CSPSTR, and MFConv to the microcrack detection performance of VUFNet. In the ablation experiments, Concat, C2f, and CBS were used to replace VUFM, CSPSTR, and MFConv, respectively. To assess the reproducibility of the observed performance improvements, each ablation configuration was independently trained and evaluated three times under the same experimental settings. The physical-blade-level dataset partition, data preprocessing procedures, training hyperparameters, and evaluation protocol were kept unchanged across all runs. The quantitative results are reported as the mean ± standard deviation in Table 3, while Figure 14 presents representative microcrack detection results.

As shown in Table 3, CSPSTR, VUFM, and MFConv all contribute positively to the performance of VUFNet. The complete model achieves the highest mAP_0.5_ and mAP_0.5:0.95_ of 97.1% and 85.6%, respectively, representing improvements of 8.3 and 7.7 percentage points over the baseline. Removing CSPSTR decreases mAP_0.5:0.95_ by 1.4 percentage points while increasing the inference speed by 3.6 fps, indicating that CSPSTR improves microcrack feature extraction at the cost of additional computational overhead. Removing VUFM results in the largest accuracy degradation, reducing mAP_0.5:0.95_ by 2.9 percentage points, which confirms its importance in effectively fusing visible-light and ultraviolet features. Removing MFConv decreases mAP_0.5:0.95_ by 0.7 percentage points but improves the speed by only 0.3 fps, demonstrating that MFConv provides additional accuracy gains with limited inference overhead. Overall, the three modules are complementary, and the complete VUFNet achieves the best balance between detection accuracy and inference speed.

Figure 14 presents microcrack detection results for selected models: (a) depicts VUFNet without VUFM, (b) shows VUFNet without CSPSTR, (c) displays VUFNet without MFConv, and (d) illustrates VUFNet. The figure indicates minimal variation between the detection results in (c) and (d), whereas the confidence levels in (a) and (b) are comparatively lower. This indicates that CSPSTR and VUFM substantially enhance the model’s detection accuracy. MFConv contributes to detection accuracy to a lesser extent. In summary, the synergistic integration of the proposed CSPSTR, VUFM, and MFConv establishes an efficient and precise microcrack detection model for turbine blades.

## 5. Conclusions

This paper addresses the challenge of detecting microcracks in aero-engine blades by proposing a VUFNet model based on multimodal fusion. Experiments employ chromic acid anodisation to enhance microcrack features, constructing visible and ultraviolet datasets. Data augmentation strategies are integrated to improve dataset diversity and scale. The VUFNet model incorporates: CSPSTR to optimise interaction between global and local features;-VUFM for deep fusion of visible and ultraviolet microcrack features; MFConv to enhance multi-scale feature extraction, improving detection accuracy in microcrack target regions. Experimental results demonstrate that VUFNet achieved an mAP_0.5:0.95_ of 85.5% and a detection speed of 52.7 fps on the held-out test set, showing improved within-domain performance over the evaluated baselines under the same experimental protocol. Ablation studies further validate the synergistic optimisation effect of VUFM, CSPSTR, and MFConv on model performance. This research provides an innovative approach for detecting microcracks in aero-engine blades and offers valuable insights for applying multimodal fusion techniques in industrial defect detection.

From a practical deployment perspective, VUFNet is being developed as the algorithmic inspection module of an automated blade-screening line. Anodised blades are transported to a fixed imaging station, where visible-light and 365 nm ultraviolet images are sequentially acquired using fixed camera–blade geometry and switchable illumination, avoiding manual repositioning. VUFNet then localises suspected microcracks and outputs the results for confirmation and maintenance-record management. Although chromic acid anodisation remains necessary, the method automates image interpretation, improves inspection consistency, and supports continuous batch screening, allowing inspectors to focus on flagged blades. End-to-end cycle time and manpower reduction are still under evaluation and are not represented by the reported inference speed.

This study has several limitations. All images were acquired from specimens belonging to a single compressor-blade type and under a controlled chromic acid anodisation and imaging protocol. Therefore, the reported results establish within-domain effectiveness but do not demonstrate generalisation across different blade geometries, materials, surface conditions, treatment parameters, imaging devices, or industrial sites. Future work will evaluate the method using additional blade types and cross-domain acquisition conditions.

It should be noted that this study focuses on the development of a multimodal detection framework, and the influence of anodisation parameters on microcrack visibility was not systematically investigated. Future work will further explore the relationship between treatment parameters (such as voltage, duration, and electrolyte concentration) and crack enhancement performance to establish an optimised and standardised pretreatment procedure.

## Figures and Tables

**Table 1:** Hyperparameters for training.

Parameters	Values
img-size	640 × 480
epochs	300
warmup	3
batch size	8
learning rate	0.01
optimiser	SGD
momentum	0.937
weight_decay	0.0005

**Table 2:** Comparison of values for each method.

Models	Images	mAP_0.5_ (%)	mAP_0.5:0.95_ (%)	Speed (fps)
Faster RCNN	Visible	68.6	55.2	22.4
Cascade RCNN	Visible	77.9	62.3	12.1
VarifocalNet	Visible	73.1	60.2	38.6
Yolov8	Visible	70.6	58.5	76.2
Yolov11	Visible	75.1	61.2	87.3
VUFNet	Visible	78.8	64.4	52.7
Faster RCNN	Ultraviolet	81.3	68.8	22.4
Cascade RCNN	Ultraviolet	89.7	74.7	12.1
VarifocalNet	Ultraviolet	84.3	72.2	38.6
Yolov8	Ultraviolet	83.0	70.3	76.2
Yolov11	Ultraviolet	87.3	73.9	87.3
VUFNet	Ultraviolet	90.3	75.7	52.7
RDFNet	Vis & Ult	92.2	79.3	49.2
CMX-B2	Vis & Ult	94.7	83.1	65.4
AsymFormer	Vis & Ult	95.5	84.3	72.3
VUFNet-Data	Vis & Ult	92.6	80.1	78.2
VUFNet-Decision	Vis & Ult	95.1	83.6	45.2
VUFNet (Ours)	Vis & Ult	96.8	85.5	52.7

**Table 3:** Impact of various improvement methods on model performance.

Models	Improved Methods			mAP_0.5_ (%)	mAP_0.5:0.95_ (%)	Speed (fps)
	CSPSTR	VUFM	MFConv			
VUFNet				88.8 ± 0.3	77.9 ± 0.4	62.0 ± 0.1
VUFNet	√			92.3 ± 0.6	81.4 ± 0.7	55.4 ± 0.2
VUFNet		√		93.3 ± 0.6	81.6 ± 0.5	59.9 ± 0.2
VUFNet			√	90.3 ± 0.5	79.2 ± 0.6	61.9 ± 0.1
VUFNet	√	√		95.5 ± 0.6	84.9 ± 0.7	52.8 ± 0.3
VUFNet	√		√	94.3 ± 0.8	82.7 ± 0.9	54.9 ± 0.3
VUFNet		√	√	94.6 ± 0.7	84.2 ± 0.9	56.1 ± 0.2
VUFNet	√	√	√	97.1 ± 0.8	85.6 ± 1.0	52.5 ± 0.3

Note: Each configuration was independently trained and evaluated three times. The dataset partition and all training settings were kept identical across the repeated runs.

## Data Availability

The data presented in this study are available on request from the corresponding author due to the internal data management regulations of the institution.

## Abbreviations

The following abbreviations are used in this manuscript:

VUFNet	Visible and ultraviolet multi-modal fusion detection network
CSPSTR	Cross-stage partial bottleneck with swin transformer
VUFM	Visible–ultraviolet multi-modal adaptive fusion module
MFConv	Multi-branch fusion convolution
W-MSA	Window-based multi-head self-attention
SW-MSA	Shifted window multi-head self-attention
MBRConv	Multi-branch re-param convolution
RDFNet	RGB-D multi-level residual feature fusion network
RFN-Nest	End-to-end residual fusion network
CMX	Cross-modal fusion framework
CM-FRM	Cross-modal feature rectification module
FFM	Feature fusion module
AsymFormer	Asymmetrical cross-modal representation learning
mAP	Mean average precision
AP	Average precision
IoU	Intersection over union
TP	True positives
FP	False positives
FN	False negatives
TN	True negatives
fps	Frames per second
MAPOD	Model-assisted probability of detection
